# Usage Durability and Comfort Properties of Socks Made from Differently Spun Modal and Micro Modal Yarns

**DOI:** 10.3390/ma16041684

**Published:** 2023-02-17

**Authors:** Antoneta Tomljenović, Juro Živičnjak, Ivan Mihaljević

**Affiliations:** Department of Materials, Fibers and Textile Testing, Faculty of Textile Technology, University of Zagreb, Prilaz baruna Filipovića 28a, 10000 Zagreb, Croatia

**Keywords:** socks, modal, micro modal, yarn type, durability, comfort, textile testing

## Abstract

Socks, being a necessary item of clothing, must be comfortable and maintain their quality throughout their life. Since the applicability of modal fibers and microfibers, as well as yarns produced using unconventional processes, in sock knitting has been insufficiently researched, this paper evaluated three groups of medium sized socks knitted in a plain single jersey pattern produced with the highest percentage of ring, rotor and air-jet spun modal or micro modal yarns of the same linear density in full plating with different textured polyamide 6.6 yarns compared to conventional cotton socks. The sock quality was evaluated through an investigation of the physical properties, wear resistance and dimensional stability, as well as the water vapor absorption, air permeability and thermal resistance using the thermal foot model before and after five repeated washing and drying cycles, according to the proposed methodology. The results showed that the fiber fineness, the structure of the differently spun yarns and the sock plain knits, the polyamide content and the implementation of the pretreatment of the socks had an influence on the obtained results. The socks made from modal and micro modal yarns differed in their properties. Compared to cotton socks, they have better comfort properties, a generally better pilling resistance and, after pretreatment, a comparable abrasion resistance.

## 1. Introduction

**Figure 1:**
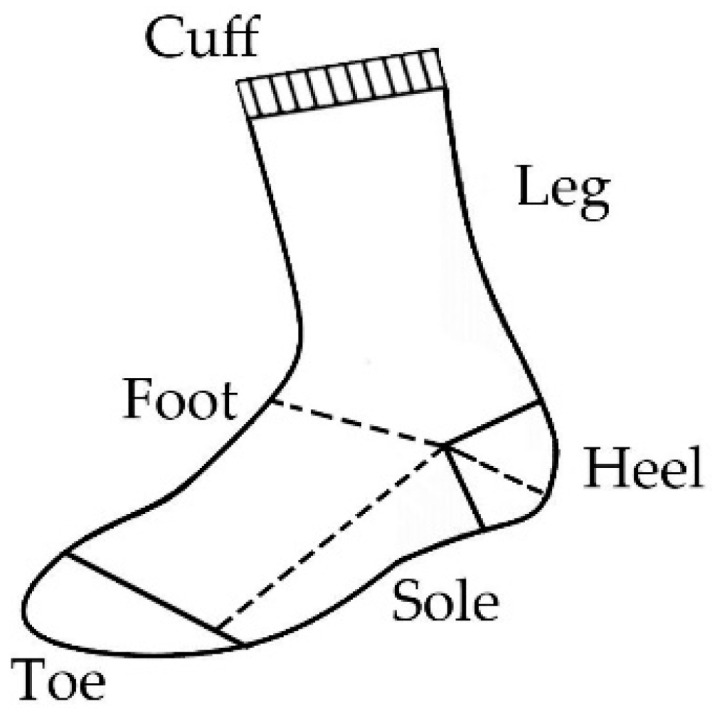
Different parts of socks.

Socks (Latin soccus) are knitted next-to-skin-type garments worn on the feet, often covering the ankle and part of the calf. They usually come in calf and over-calf lengths and are worn inside shoes. They consist of the top part, the part that covers the leg (leg part) and the part that covers the foot (foot part). More precisely, the main parts of socks (Figure 1) are the cuff, leg, heel, sole, toe and foot [1,2].

Socks have to fulfill high requirements for their usage durability and comfort. Therefore, it is very important to select the right fibers and yarns for their production [3]. Socks come in a variety of fibers and fiber combinations. Usually, casual socks are made from a high percentage of cotton or wool to provide softness and comfort. They are blended with polyamide and/or elastane to improve the fit, durability and shrink resistance. Occasionally, acrylic, polyester, polypropylene or luxury fibers such as silk, linen, cashmere or mohair are also added to improve the sock properties [4]. Man-made artificial fibers made from cellulose (e.g., viscose, modal and lyocell) offer a silky handle, exceptional contact comfort and better hydrophilicity than cotton and play an important role in the textile market for lingerie items worn in direct contact with the skin [5]. Their applicability in the knitting of socks, especially for modal fibers and microfibers, is still insufficiently researched. The comparison of the modal and cotton fibers properties, relevant to this study, is shown in Table 1.

Many scientific papers have analyzed the improvement of the properties of socks using different types of fibers, e.g., cotton, wool, acrylic or their blends, and either blended or not with polyester, polyamide and/or elastane [1,5,10,11,12,13,14,15]. Few authors include viscose and bamboo, soybean, flax [16,17,18,19] and even reclaimed the cotton fibers [20,21] in the analysis of the sock knits. A group of authors used fibers such as modal, micro modal, viscose, bamboo, soybean and chitosan to produce plain single jersey sock samples to compare their comfort-related properties with those of cotton socks [22,23]. However, it should be noted that polyamide and elastane were not added, in contrast to the commercial production conditions for socks. The results indicated that the fiber type, together with the moisture regain and fabric thickness, affected the comfort properties in the socks knits, with modal, viscose, chitosan and seacell fabrics performing relatively better than the others. Compared to micro modal sock fabrics, the modal sample was found to have a lower thermal resistance, suggesting that microfiber fabrics have a lower thermal conductivity and, thus, better insulation properties.

Different types of yarns are used for socks, the most important of which are the main yarn and the plating yarn. Socks are usually knitted from single spun main yarns using a conventional ring spinning system. Unconventional rotor and air-jet spinning technologies result in yarns with different structures and properties [24]. Numerous comparative studies have been published on the properties of ring, rotor and air-jet spun yarns and the knitted fabrics made from them [24,25,26,27,28]. Air-jet spun yarns exhibit a lower hairiness and fewer irregularities because they have a uniformly distributed layer of wrapped fibers compared to yarns produced with rotor and ring spinning systems. Consequently, fabrics made from air-jet spun yarns are known to have a very smooth handle and are less prone to surface pilling. Ring spun yarns are hairier but more compactly structured and have a higher tenacity than rotor and air-jet spun yarns. Therefore, knitted fabrics made from ring spun yarns have a better abrasion resistance [29]. However, the applicability of unconventionally spun yarns in the knitting of socks has not yet been sufficiently researched. A few published studies have reported the effect of unconventionally spun main yarn on the properties of sock fabrics, such as a study aimed at determining the relative effect of the wool fiber fineness, yarn type (ring two-ply conventional high twist, unconventional low twist and single conventional) and fabric structure on the thermal and moisture transfer properties, cyclic compression and friction properties of sock fabrics [30,31,32]. It was found that the effect of the yarn type was directly related to the physical properties of the fabric, especially the fabric thickness. The thermal resistance of the fabrics composed of ring two-ply yarns with a low twist was higher [31]. The thermal comfort and physical properties—as well as the air permeability, bursting strength and pilling grade of the socks made from two types of cotton open-end rotor spun yarns, one made from a blend of reclaimed cotton fibers and polyester, and the other made from cotton fibers—were investigated along with the influence of elastane [20,21].

The knitted structures used for socks must have an adequate elasticity and fit for the feet and legs. The very stretchy rib knit structures (e.g., rib 1 × 1, rib 2 × 2) are generally used for the top part and rarely for the leg part of casual socks, while the plain knit stich is used in the foot part [1,4]. In this case, due to the advantageous properties contributing to socks, the elasticity in the leg and foot parts is provided using synthetic highly elastic plating yarns. Usually, polyester, polyamide and/or elastane yarns are used [3,20].

It is well known that the air, moisture and heat transfer through the garment are the most important factors in clothing comfort [19]. Socks should provide more comfort than other garments as there is less air circulation in socks and in shoes [2,33]. Air permeability is an important comfort factor for transporting moisture or vapor from the skin. Air permeability is a function of the thickness and surface porosity of the knit [33,34] and ensures the breathability of the socks.

The moisture regain is a parameter that quantifies the hydrophilicity of a textile material and depends on the relative humidity and temperature. The swelling properties are related to the moisture regain; the more hydroscopic a fiber is, the more it swells with water [35]. The absorption capacity of textiles has been reported to be influenced by many factors, including the fiber chemistry and morphology, fabric thickness and structure, yarn type, surface properties of the fibers and fabrics, size and shape of the yarn and fiber interstices and finishing treatments of the fibers, yarns and fabrics [30].

The thermal resistance of textiles is influenced by the fiber conductivity, fabric porosity and fabric structure [16]. The thickness of the fabric and the volume of the trapped air generally determine the thermal resistance of the sock fabric under static conditions, with less effect from the fiber itself. Thinner fabric traps less air and is, therefore, typically less insulating than thicker fabric. The effect of the fibers on the thermal resistance is usually limited due to their packing density in the fabric structure, which contributes to the volume of the trapped air [30,36]. In the case of socks, the extensibility of the knitted fabric at the wearer’s foot must also be taken into account [3]. Given the increasing demand for comfortable clothing, numerous studies have been conducted on the thermal resistance of fabrics. Thermal foot manikins, available since the late 1990s, have been used primarily to study the thermal resistance of footwear. Despite the important contribution of the sock to the thermal protection provided by the footwear, there are significantly fewer studies investigating the thermal resistance of sock fabrics or socks alone [30]. The thermal resistance of socks has been measured under steady state conditions using the thermal foot model [3,16,36] and the sock-shaped hot plate [22,23]. The thermal resistance of the sock fabrics was measured by a standardized method according to EN ISO 11092:2014 using a sweating guarded hot plate [2,30,37]. Since this test requires rectangular seamless samples measuring 510 × 510 mm, the thermal resistance of the sock fabrics was also measured on the smaller knit samples using the Permetest or Alambeta tester (equivalent to ISO 8301:1991) [16,21] and calculated as the quotient of the fabric thickness and thermal conductivity parameters of the sample [17,18,19]. Several studies have been conducted on the wearing of socks in humans, generally focusing on the perception or measurement of the thermal comfort and foot dryness directly on the wearer’s feet [14,38].

Socks should maintain their quality throughout their lifetime. They must meet high wear resistance requirements, especially a higher abrasion resistance and a lower propensity to surface pilling. Abrasion, which is an unavoidable problem, usually occurs on the heel, sole and toes of the socks. The sock rubs in the shoes, slippers or even on the floor. In the first stage of abrasion, the small pill balls get tangled due to the loose fibers that shed from the knitted surface when worn and washed, resulting in an undesirable appearance and unpleasant hassle. Eventually, the fibers that bind the pills to the surface break down and a hole or thinning occurs [4,15]. The abrasion resistance and propensity to surface pilling of sock knits are influenced by many factors, such as the type of fibers and blends, the structure of the yarns, the construction of the knit and the finish [4,17,39]. Various researchers have studied the abrasion resistance and surface pilling tendency [3,4,15,20,29,33] of sock knits. It has been found that the abrasion resistance of socks knitted in a single jersey structure can be increased by a number of measures: the use of yarns with a higher linear density, yarns with a higher twist coefficient and folded yarns, the addition of polyester fibers in blends and the addition of elastic yarns (polyamide or elastane filaments) to the structure [4,15,33]. In recent research, highly functional socks were knitted in the plain single jersey pattern in full plating using three-ply and two-ply twisted yarns composed of various high-performance fibers with PCMs, insect repellent, bio-ceramic, silver and carbon additives [2,37]. The use of spiral auxiliary yarns consisting of various combinations of high-performance para-aramid, elastomeric, polyamide, polyester and conventional cotton fibers has also been investigated for the development of abrasion-resistant socks [13]. When evaluating the sock-to-skin friction, the plain single jersey structures knitted with two cotton yarns were found to be the most suitable for running socks [35]. A review of the literature shows that no studies were found on the wear resistance of socks knitted with three single spun yarns in full plating using the plain knit stitch.

Dimensional stability is one of the basic requirements for socks [11]. The leg and sole parts can undergo major shrinkage after washing, which affects the usability of the socks due to size mismatching problems [5,10]. However, most published work has been concerned with the dimensional stability of knitted fabrics rather than socks [11]. The dimensional properties of single jersey knitted fabrics are mainly influenced by the constituent fibers, yarn properties, knitting machine variables, processing and finishing [40]. The shrinkage of knitted fabrics was studied considering the knit properties and wet treatments. It was found that fabric shrinkage is strongly influenced by the type of yarn and fiber blend [41]. The dimensional differences between knitted jersey fabrics made from open-end rotor and ring spun yarns were also investigated. The fabric made from open-end yarns had a relatively good dimensional stability [42]. Studies concerning the sock dimensional stability are very few. The effects of three different parameters, namely the linear density of yarn, the loop length and the construction, on the dimensional stability of 100% cotton socks during wet processing were compared. It was found that the loop length had a significant inverse effect on the dimensional stability of the socks [5]. Cotton/polyamide 70/30 and cotton/polyamide/elastane (79/20/1) socks were found to shrink significantly after the first wash. The second wash had no significant effect on the dimensional variations, as the socks had already assumed their fully relaxed dimensions [10]. It was found that, as the elastane content increases, the shrinkage of the socks decreases [11], and that the drying temperature and the external force during the drying time also have a great influence on the dimensional change of the socks [43].

The number of European standards for the testing of knitwear and socks are low [3]. Since the properties of the socks change after domestic care, and the pretreatment of the samples in some standardized test methods is only given as an option without an obligation for use, it is necessary to expand the research in the field for a quality assessment of socks. It should be noted that there is no standard or official procedure specifically for determining the dimensional stability of socks, and there are very few studies on the thermal resistance of socks measured on the thermal foot model alone.

The literature review showed that the applicability of modal fibers and microfibers, as well as the unconventionally spun yarns made from them, in knitting socks has not been sufficiently researched. Additionally, the analysis of the usage durability and comfort-related properties of these kind of socks produced under commercial production conditions with the addition of polyamide and elastane plating yarns, especially those knitted with three single spun yarns in full plating, was not founded in the literature. There are no research results on the influence of conventional cotton yarns used in combination, as well as polyamide plating yarns of different properties. Therefore, this work evaluated three groups of socks knitted in single jersey pattern with the highest percentage of single ring, rotor and air-jet spun modal or micro modal yarns of the same linear density in full plating with different textured polyamide 6.6 yarns. The influence of the different yarn combinations in socks produced under the same conditions was investigated in comparison to the conventional sock samples produced from cotton yarns. The evaluation of the sock quality was carried out through an investigation of the basic physical properties, wear resistance and dimensional stability, as well as the water vapor absorption, air permeability and thermal resistance using the thermal foot model before and after five repeated washing and drying cycles, according to the proposed methodology.

In this context, the aim of this work was to determine the applicability of modal and micro modal yarns for the production of socks, to determine the influence of the differently spun main yarn types and the polyamide plating yarns used on the tested sock properties and to confirm the applicability of the proposed methodology for the evaluation of the sock quality, which includes the implementation of a pretreatment.

## 2. Materials and Methods

### 2.1. Sock Production and the Yarns Used

Six differently spun yarns with a nominal linear density of 20 tex were selected as the main yarns for the knitting of socks—conventional single ring spun, unconventional single rotor and air-jet spun yarns, all composed of bright modal staple fibers (MD) with a linear density of 1.3 dtex or modal microfibers (MMD) of 1.0 dtex and a length of 38/40 mm.

- The ring spun yarns (Ri) of the modal and micro modal fibers were produced using the following process: the preparation process (opening, blending and mixing), carding process, spinning preparation (drawing, pre-spinning and ring spinning), winding and cleaning. The yarns were spun on a Zinser 351 ring spinning machine (ring diameter: 42 mm, ring type: f2, spindle speed: 16,500 min^−1^), wound up and cleaned on an Autoconer X5. The number of twists for the modal ring spun yarns was 746 m^−1^ and 734 m^−1^ for the micro modal spun yarns.
- The rotor spun yarns (Ro) from the modal and micro modal fibers were produced using the fiber preparation process (opening and blending), carding, spinning preparation (drawing) and rotor spinning using a Schlafhorst A8 rotor spinning machine with a rotor diameter of 33 mm. The nominal twist number of the rotor yarns calculated from the rotor speed was 750 twists per meter.
- The air-jet spun yarns (Ai) from the modal and micro modal fibers were produced using the preparation process (opening and blending), the carding process, spinning preparation (three drawing passages) and air-jet spinning on a Rieter J20 machine with an inner spindle diameter of 1.2 mm. The air-jet yarn twists were determined according to the high pressure of the air in the rotating vortex at 0.6 MPa [27].

**Figure 2:**
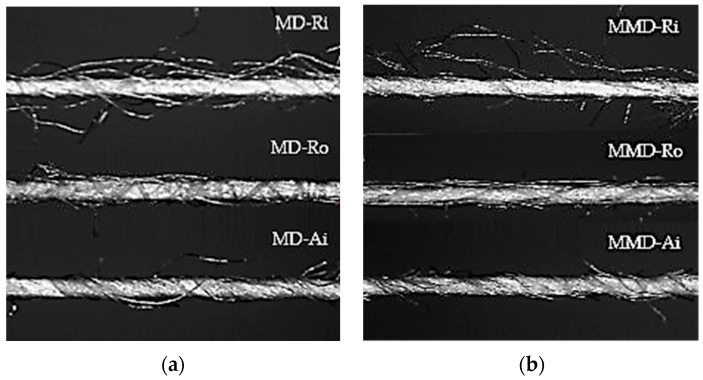
Optical microscopy images of the yarns’ longitudinal view (magnification 60×): (**a**) modal ring (MD-Ri), rotor (MD-Ro) and air-jet (MD-Ai) spun yarns; (**b**) micro modal ring (MMD-Ri), rotor (MMD-Ro) and air-jet (MMD-Ai) spun yarns.

Figure 2 shows the longitudinal view with the characteristic surface structure and twists for the modal and micro modal ring, rotor and air-jet spun yarns used.

Two textured multifilament polyamide 6.6 plating yarns with different properties (PA 6.6 (1); designation: PA 6.6 156 dtex f 42 and PA 6.6 (2); designation: PA 6.6 220 dtex f 68) and one single Lycra with a linear density of 54 tex (usually used in commercial production) were selected as the plating yarns. A polyamide plating yarn was knit into the toe, heel, foot, leg and top part of the socks for reinforcement and support, along with the three single spun yarns in each knit course. An additional single Lycra yarn was knitted into the cuff at the top of the socks only to keep them from falling.

With the aim of determining the influence of the differently spun main yarn types, the selected polyamide plating yarns and the conventional cotton yarn used in combination with the sock properties, three groups (A, B and C) of medium sized calf length socks were designed and produced.

- Sock group A, knitted with three single spun modal or micro modal yarns of the same type (spun by a ring, rotor or air-jet spinning system) in full plating with a polyamide PA 6.6 (1) yarn
- Sock group B, knitted with three single spun modal or micro modal yarns of the same type (spun by a ring, rotor or air-jet spinning system) in full plating with a polyamide PA 6.6 (2) yarn
- Sock group C, in which one of the single spun modal or micro modal yarns was replaced by a coarser cotton ring spun yarn (CO-Ri (2). This group of socks was, thus, knitted with two single spun modal or micro modal yarns of the same type (spun by a ring, rotor or air-jet spinning system) in combination with a cotton ring spun yarn in full plating with a polyamide PA 6.6 (2) yarn.

The influence of the different yarn combinations in the socks was investigated in comparison to the conventional sock samples (of groups A, B and C) produced under the same conditions using a single cotton ring spun yarn (CO-Ri (1)) with a nominal linear density of 20 tex. The properties of all the yarns used for the knitting of socks are given in Table 2.

A description of the socks produced, including the values of the fiber content in the specific parts of the sock knitted structure, is given in Table 3.

**Figure 3:**
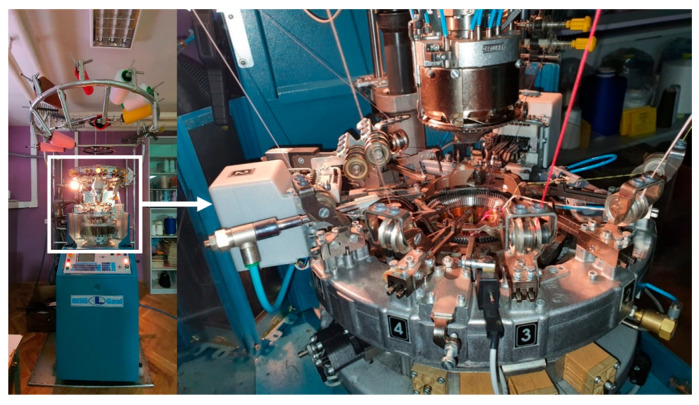
Automatic sock knitting machine, Lonati, Goal FL 626.

All the sock samples of the same size (EU 42) were knitted using a Lonati automatic sock knitting machine with an E9 gauge and a cylinder diameter of 95 mm (3 ¾’’) with 108 needles and two knitting systems (Figure 3). After sewing the toes, the socks were ironed and stabilized on flat leg forms using an industrial Cortese ironing machine under the following conditions: pressing for 4 s and steaming (saturated steam at 3.17 kg/m^3^) with a pressure of 0.7 bar for 6 s at a temperature of 110 °C. The plain single jersey pattern was used in the foot and leg part, and a 1 × 1 rib structure was used in the cuff of the socks. The toe seams were placed high over the toes, the toe and heel parts were smooth, and the square heel shape were made.

### 2.2. Socks Evaluation Methodology

In this paper, the usage durability of socks was evaluated through an investigation of the wear resistance, dimensional stability and comfort-related properties, as well as the water vapor absorption, air permeability and thermal resistance. In addition, the physical properties of the socks consisting of the density parameters, mass and thickness were measured to define their influence on the mentioned properties.

**Figure 4:**
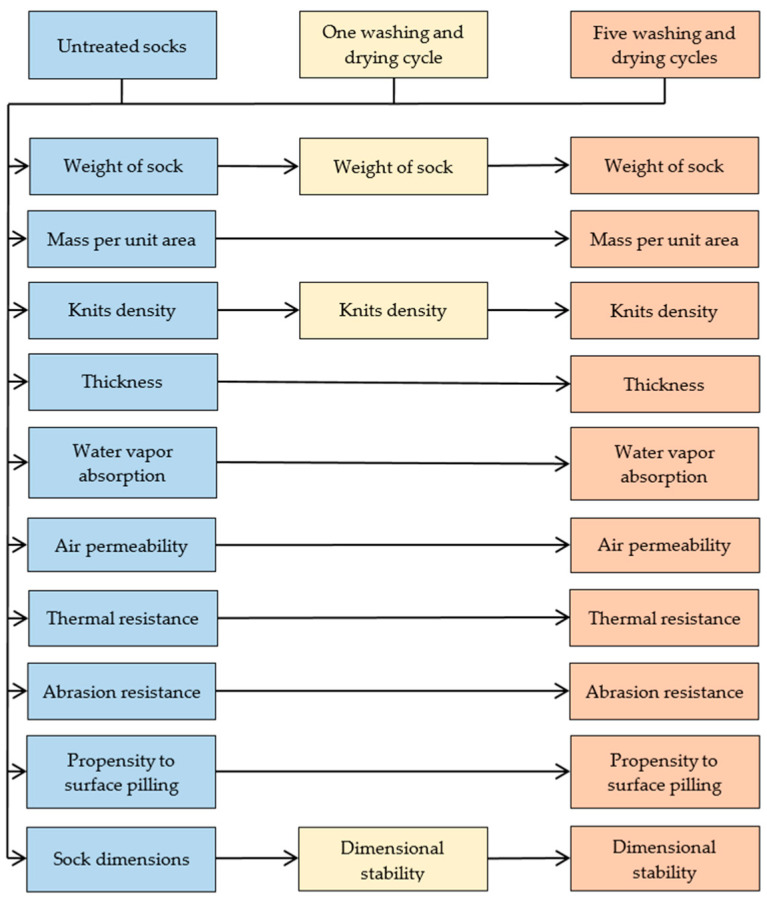
Schematic view of the sock quality evaluation methodology that includes the selected performance properties.

Since the properties of socks change after domestic care, and the pretreatment of the samples is only indicated in some standardized test methods as an option without an obligation for use, all the tested properties were measured before and after one and the five repeated washing and drying cycles of the socks, according to the proposed methodology (Figure 4). The socks were washed according to the procedure 3M of EN ISO 6330:2012 at a temperature of 30 °C with mild agitation during heating, washing and rinsing using a non-phosphate ECE reference detergent without an optical brightener in the Electrolux Wascator FOM71 CLS. After each wash cycle, the socks were line dried in the open air (procedure A).

**Figure 5:**
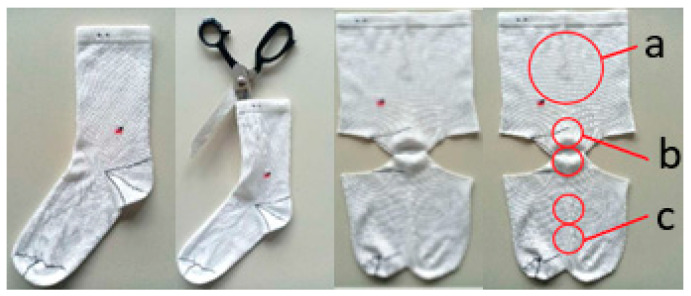
Cutting the socks and the sampling of the test specimens: (**a**) for testing the propensity to surface pilling, mass per unit area and water vapor absorption; (**b**,**c**) for testing the abrasion resistance.

Prior to measurement, the untreated and the pretreated sock samples were conditioned on a flat surface for at least 24 h in a standard atmosphere with a temperature of 20 ± 2 °C and a relative humidity of 65 ± 4%. For all the tests on the sock knits, the socks were cut open and sampled as necessary, as shown in Figure 5.

#### 2.2.1. Sock Physical Properties Measurements

The following physical properties of the socks were determined:

- The weight of one sock expressed in grams
- The sock plain knit mass per unit area according to the EN 12127:2003 expressed in g/m^2^
- The sock plain knit thickness according to the EN ISO 5084:2003 expressed in millimeters, using the Hess MBV GmbH thickness tester 2000-U
- The sock plain knit density parameters consisting of the number of wales/cm, courses/cm and stitches/cm^2^, according to the EN 14971:2008

The number of measurements was five for the mass and density tests and ten for the thickness test, with the mean values given as the result.

#### 2.2.2. Sock Usage Properties Measurements

**Figure 6:**
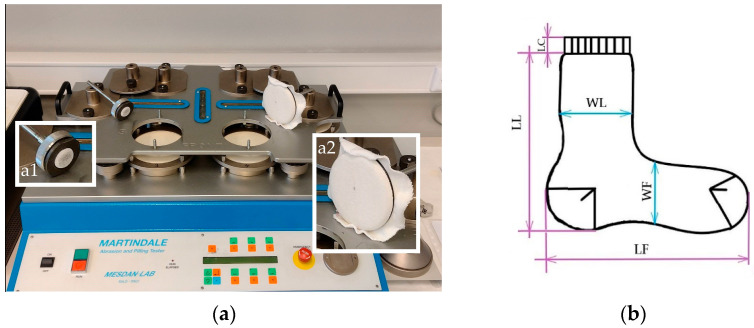
Evaluation of the usage properties of socks: (**a**) the device for determining the wear resistance of sock knits, Mesdan-Lab Martindale abrasion and pilling tester 2561 E, with (a1) the specimen holder for the abrasion resistance test and (a2) the specimen holder for the pilling test; (**b**) the measurement scheme for the dimensions of the socks: length of cuff, leg and foot part (LC, LL and LF); width of leg and foot part (WL and WF).

The wear resistance of the socks was determined by measuring the following properties:

- The sock plain knit abrasion resistance was measured using the Mesdan-Lab Martindale abrasion and pilling tester (Figure 6a) in accordance with the EN 13770:2002, method 1, through the determination of the specimen breakdown, where the plain knit specimens were abraded against the reference wool abrasive fabric with a cyclic planar motion in the form of a Lissajous figure. The SDC Enterprises Limited UK Martindale woven wool abradant with a mass per unit area of 250 g/m^2^, as specified in the EN ISO 12947-1:1998+AC:2006, was used for the test. Two circular specimens with a diameter of 38 ± 5 mm were taken from the heel and two from the sole of the socks (Figure 5). During the test, the specimens were stretched over a flattened rubber surface of the holders and loaded with the corresponding weight of 12 kPa. The endpoint was defined as the occurrence of the specimen breakage (the breakage of the thread in the knitted structure, usually resulting in a hole) or significant thinning (wear of the main spun yarns), which was periodically checked. During the inspection, the pills were removed using sharp scissors with curved blades. The number of rubs until the endpoint was reached was recorded.
- The sock plain knit propensity to surface pilling was measured according to the EN ISO 12945-2:2020 using the modified Martindale method (Figure 6a). Three specimens were rubbed according to the Lissajous figure against the same reference wool abradant loaded with the corresponding weight of 415 g. The specimens were cut in a circular shape with a diameter of 140 ± 5 mm and taken from the socks leg part (Figure 5). During the test, the specimens were visually assessed after 125, 500, 1000, 2000, 5000 and 7000 pilling rubs, according to the EN ISO 12945-4:2020, with grades 1 to 5 corresponding to the appropriate pilling degrees. Each specimen was evaluated separately by three experts and the result was expressed as a mean value.

The EN ISO 3759:2011 and EN ISO 5077:2008 standards, normally used for ordinary knitted fabrics, was adopted to determine the dimensional variation of the socks after one and five consecutive washing and drying cycles. The length and width dimensions, measured at the top, foot and leg of the untreated and pretreated socks were determined according to the specification shown in Figure 6b. The percentage change in the length and width of the socks was calculated, and for the condition of whether the dimension decreased, the shrinkage was expressed as a minus.

#### 2.2.3. Socks Comfort Properties Measurements

**Figure 7:**
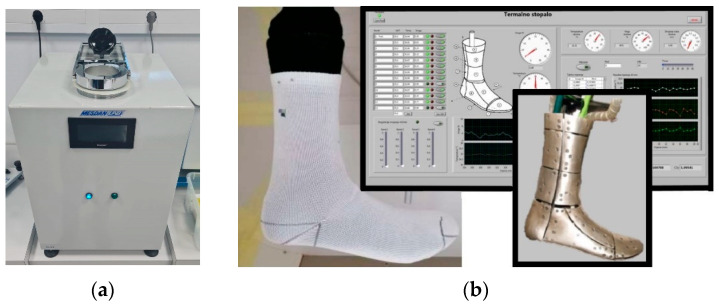
Evaluation of the comfort-related properties of socks: (**a**) the device for determining the air permeability of the sock knits, Air Tronic Mesdan S.p.A.; (**b**) the thermal foot manikin system.

The following comfort-related properties of the socks were studied:

- The water vapor absorption of the sock plain knits was determined according to the ASTM D 2654-89a. The circular specimens of the plain knits with an area of 100 cm^2^ were cut from the leg part of the conditioned socks, weighed, then dried in an oven at 105 ± 2 °C for 24 h and reweighed. The difference between the mass of the conditioned and the mass of the oven-dried specimens was calculated as the moisture regain and expressed as a percentage. The mean value of three measurements was given as the result.
- The permeability of the sock plain knits to air was determined according to the EN ISO 9237:1995 using the air permeability tester shown in Figure 7a. The arithmetic mean of the individual air flow readings in a test area of 5 cm^2^ and a pressure drop of 100 Pa was noted. The air permeability was calculated according to Equation (1)
  (1) $$R=\frac{\bar{qv}}{A}\cdot 167$$
  where *qv* is the arithmetic mean of the air flow expressed in dm^3^/min, *A* is the test surface area expressed in cm^2^ and 167 is the conversion factor from dm^3^/min cm^2^ to mm/s.
- The thermal resistance of the socks was defined as the ability of the socks to resist the heat flow through their knitted structure, using the thermal foot manikin system (Figure 7b). It consisted of the thermal foot (EU size 42), a stainless steel support structure, shock absorbers and a heating subsystem. The heating subsystem, controlled by the personal computer was connected to the thermal foot using highly flexible cables. The thermal foot was divided into 13 silver alloy surface segments that were independently heated to a temperature of 35 ± 0.5 °C. Since the heaters and temperature sensors were installed in each segment, the thermal resistance on each segment or the total resistance could be determined using a special algorithm. The two upper segments were excluded from the measurement, so the total measurement area at the thermal foot was 88.190 mm^2^. The apparatus constant (*R*_*ct*0_) needed to be determined first under the defined environmental conditions of 20 ± 2 °C air temperature, 65 ± 4% relative humidity, an air speed of 1 m/s. The sock to be tested was then placed on thermal foot and the total thermal resistance (*R_ctt_*) of the apparatus and the sock was measured. The thermal resistance of the tested sock sample (*R_ct_*) was calculated from the difference between *R_ctt_* and *R*_*ct*0_ according to Equation (2)
  *R_ct_* = *R_ctt_* − *R_ct_*_0_ (2)
  where *R_ct_* is the thermal resistance of the tested sock, *R_ctt_* is the total thermal resistance of the apparatus and the sock and *R*_*ct*0_ is the thermal resistance of the apparatus (thermal foot), all in m^2^ °C/W. As a result, the mean value of the measurements on the three sock samples of the same group was provided.

## 3. Results and Discussion

The results include the measured physical, usage and comfort properties of the designed socks. The influence of the different spun main yarn types, polyamide plating yarns and conventional cotton yarns in combination with the sock properties was discussed.

### 3.1. Sock Physical Properties

**Figure 8:**
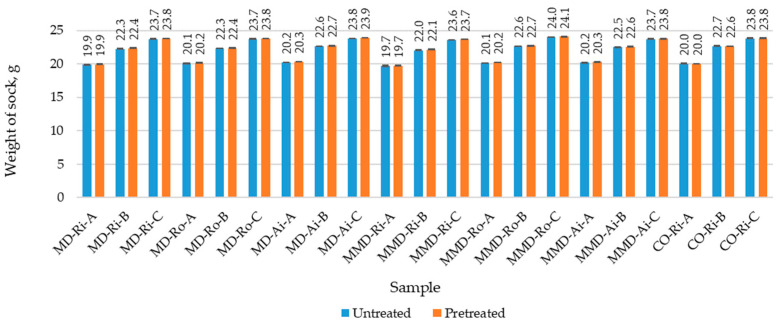
Weight of the untreated and pretreated sock samples determined before and after five repeated washing and drying cycles (where MD—modal fibers, MMD—micro modal fibers, CO—cotton fibers; Ri—ring spun yarn, Ro—rotor spun yarn, Ai—air-jet spun yarn; A, B, C—sock group).

**Figure 9:**
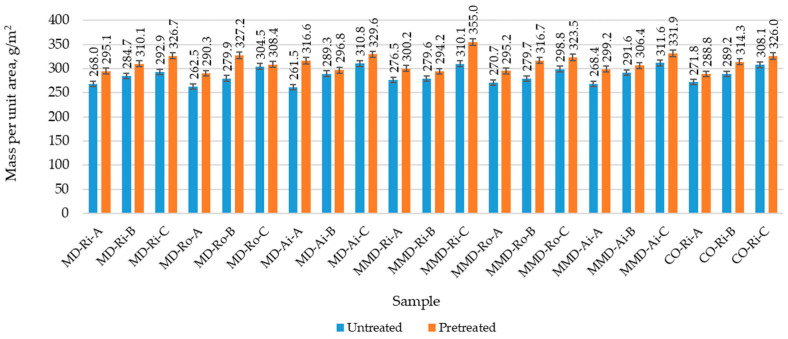
Mass per unit area of the untreated and pretreated sock plain knit samples determined before and after five repeated washing and drying cycles of the socks (where MD—modal fibers, MMD—micro modal fibers, CO—cotton fibers; Ri—ring spun yarn, Ro—rotor spun yarn, Ai—air-jet spun yarn; A, B, C—sock group).

**Figure 10:**
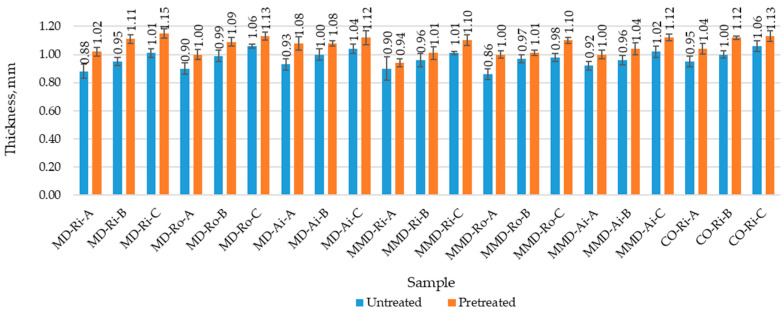
Thickness of the untreated and pretreated sock plain knit samples determined before and after five repeated washing and drying cycles of the socks (where MD—modal fibers, MMD—micro modal fibers, CO—cotton fibers; Ri—ring spun yarn, Ro—rotor spun yarn, Ai—air-jet spun yarn; A, B, C—sock group).

The results of the measured physical properties of the untreated and pretreated sock samples (determined before and after the five repeated washing and drying cycles) with the corresponding standard deviation are shown in Figure 8, Figure 9 and Figure 10 and in Table 4. Table 5 contains a comparison of the mean values of the physical properties of the untreated and pretreated socks (made from differently spun modal or micro modal yarn) calculated for groups A, B and C with the corresponding standard deviation.

As can be seen in Figure 8 and Table 5, all the sock samples of group A made from modal and micro modal yarns had a lower weight than the socks in groups B and C. The values for the untreated socks were 20.0 g, 22.4 g and 23.8 g, respectively, with almost no variation within the same group compared to the cotton socks made with the highest percentage of ring yarns. This indicates a high quality in the sock production and suggests that the coarser polyamide plating yarn PA 6.6 (2) used in sock groups B and C and the cotton ring spun yarn CO-Ri (2) used in group C change the weight of the socks and their structure in such a way that these socks become thicker and heavier.

Therefore, the lowest values of the thickness and mass per unit area of the untreated sock plain knits made from modal and micro modal yarns were determined for the sock samples in group A and the highest values for the sock samples in group C (Figure 9 and Figure 10). According to the results presented in Table 5, despite the minor variations within the sock groups, there were small differences between the mean thickness values calculated for the same group of socks (A, B and C) made from modal and micro modal yarns (Table 5).

According to the previously published results, the number of modal fibers in the yarn cross-section was between 155 and 156, while the number of microfibers in the yarn cross-section was between 200 to 202. The number of twists was also uniform for all the yarn types; the twist coefficient ranged from 3.280 to 3.350 m^−1^ tex ^0.5^ [27]. However, the values of the overall unevenness of the ring, rotor and air-jet spun modal yarns (10.21%, 13.95% and 12.33%, respectively) were higher than the same values of the micro modal yarns (9.67%, 12.69% and 12.12%, respectively). The values of the hairiness of the ring, rotor and air-jet spun modal yarns (6.09, 4.34 and 3.71) were also higher than those of the micro modal yarns (5.28, 4.08 and 3.56). Thus, the hairiness and the overall unevenness of the yarn depend mainly on the spinning technique and the fineness of the fiber. This led us to the conclusion that a more uniform structure of micro modal yarns has a major influence on the slightly lower mean values of the determined thickness (Table 5) for the same sock group.

As can be seen in Figure 10 and Table 5, the mass per unit area results showed a greater variability within and between the same sock groups. This can be explained by the differences in the yarn structure as well as in the structure of the plain knit of the socks, which have a major effect on the mass per unit area of the fabric. Despite the fact that all the socks were knitted under the same conditions, the number of courses/cm of the untreated socks was uneven, which consequently led to uneven changes in the number of stitches/cm^2^ in the pretreated samples (Table 4).

After one and five repeated washing and drying cycles, as shown in Figure 8, the weight of the socks made from the highest percentage of differently spun modal, micro modal and conventional cotton yarns did not change significantly. It remained almost the same for the cotton socks, while a minimal increase of up to 0.5% was observed for the socks made from MD and MMD yarns. This can be explained by the full relaxation of the socks after the water pretreatment and the presence of the structural differences, owning to which they have a better ability for absorbing moisture, as shown in Table 1.

From the results presented in Table 4 for the density of the sock plain knits, it can be seen that the number of wales per cm, courses per cm and stitches per cm^2^ increased mainly from washing, with most of the changes occurring after one and remaining the same after five pretreatment cycles. It is well known that a knitted fabric is brought to a tension-free state, i.e., a state with minimal energy, using a full relaxation treatment that includes a wet treatment with mechanical agitation and drying [20]. Consequently, after pretreatment, when a sock knit structure approaches its minimum energy, the fabric width and length shrinkage occurs. The increase in the density values of the sock plain knits achieved after the five pretreatment cycles resulted from the sock shrinkage, which was higher in the length of the socks.

After the five repeated washing and drying cycles, the mass per unit area and the thickness largely reflected the density parameters of the sock plain knits. Due to the shrinkage potential of the socks, the socks became more voluminous as the relaxation process progressed, resulting in an increase in the mass per unit area and the thickness of up to 16%, with the same traceability of the results.

### 3.2. Socks Usage Properties

**Figure 11:**
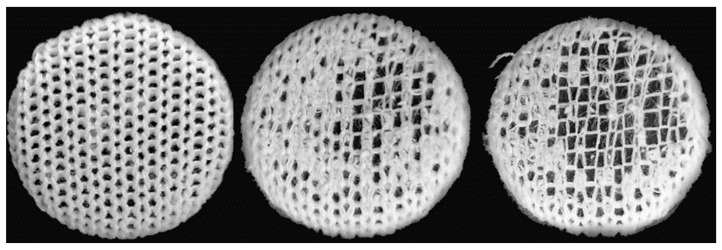
Surface appearance of the untreated heel plain knit sample MD-Ai-A, before, during and at the end of the abrasion resistance test (determined at 0, 8000 and 12,000 abrasion rubs).

In this study, the abrasion resistance was estimated from the number of abrasion rubs to the significant thinning of one component in all the untreated and pretreated sock plain knits tested, where the spun staple yarns wore out and the base of the polyamide multifilament plating yarns remained, as shown in Figure 11. The polyamide filaments were more difficult to liberate from the fabric structure and were generally considered highly abrasion resistant, while the cotton and modal fibers were moderately abrasion resistant [2,44].

**Figure 12:**
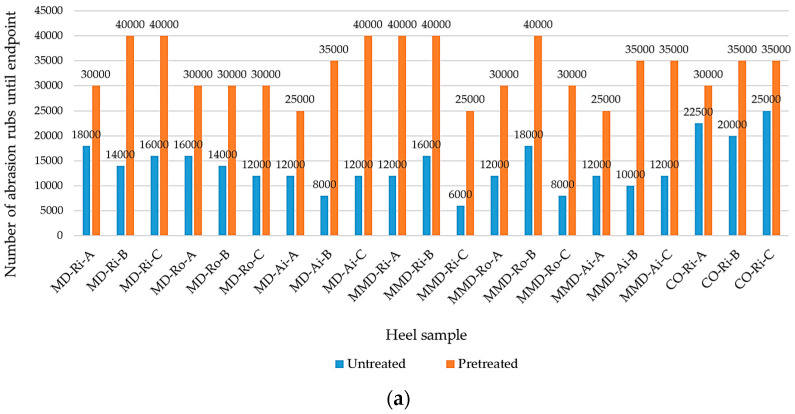

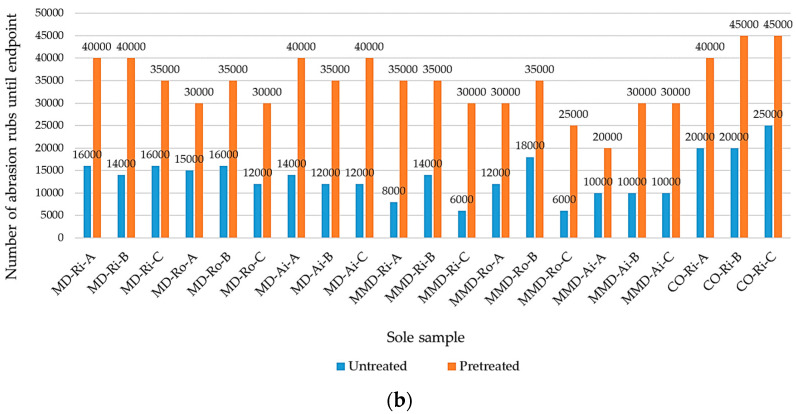
Abrasion resistance of the untreated and pretreated plain knits sampled from (**a**) the heel of the socks; (**b**) the sole of the socks, determined before and after five repeated washing and drying cycles (where MD—modal fibers, MMD—micro modal fibers, CO—cotton fibers; Ri—ring spun yarn, Ro—rotor spun yarn, Ai—air-jet spun yarn; A, B, C—sock group).

The endpoints reached in the plain knits sampled from the heels and soles of the tested socks, are shown in Figure 12a, b. The untreated sock knits made from modal ring spun yarns exhibited better abrasion resistance than those made from rotor and air-jet modal spun yarns, which was particularly noticeable in the sock heel (Figure 12a). Ring spun yarns are hairier but more compactly structured, which means that the fibers are more twisted at their surface than in rotor yarns (Figure 2), which do not promote slight fiber wear. This may also be related to the fact that ring spun yarns have a higher breaking strength, elongation at break, tenacity and work of rupture (Table 2) than air-jet and rotor spun yarns, which means that they can better withstand repeated distortion. In general, for the untreated socks samples in group A produced from modal yarns in full plating with textured polyamide yarn PA 6.6 (1), the specimen breakdown occurred at the highest values of the abrasion rubs recorded. Despite the fact that the polyamide content was increased in sock groups B and C (Table 3), it was found that this had no effect on the improvement of the abrasion resistance. Increasing the fineness of the fibers further changes the properties of sock knits. The use of microfibers in the production of yarns leads to an increase in the number of fibers in the cross-section with a higher cohesion, which—together with the addition of PA 6.6 (2) elastic plating yarn with a higher linear density, breaking force and work at rupture (Table 2)—primarily leads to a better abrasion resistance determined in the untreated sock group B (from ring and rotor spun yarns). In the untreated sock group C, where one of the single spun modal or micro modal yarns was replaced by a cotton ring spun yarn CO-Ri (2), no improvement in the abrasion resistance was observed. When comparing the untreated socks, lower abrasion resistance values were obtained for MD and MMD socks than for conventional cotton socks (especially those with a thicker and heavier structure). The determined abrasion resistance was lowest for the untreated sock plain knits with the highest proportion of micro modal yarns.

From the results shown in Figure 12a,b, it can be seen that the abrasion resistance increased greatly after the five washing and drying cycles for all the sock samples tested. The pretreated socks were found to increase the abrasion resistance by up to 77% for the socks made from modal fibers, 80% for the socks made from micro modal fibers and up to 56% for cotton socks. Overall, the abrasion resistance of the pretreated socks made from modal and, in most cases, micro modal yarns was comparable to that of conventional cotton socks, with significant thinning occurring within the interval of 30,000 to 45,000 abrasion rubs. During the wet pretreatment, the fibers stick to the surface of the knitted fabric, so that the fabric reaches a tighter state and the movement of the fibers within the yarn is limited. The result is a more stable, thicker and voluminous sock structure. Another parameter affecting the abrasion is the stitch density of the sock plain knit (see Section 3.1). The more stitches per unit area in the fabric, the lower the force on each thread. The plain knits with a tighter structure of socks had a higher abrasion resistance. It can be concluded that the structural abrasion of the pretreated sock samples was lower than the surface abrasion, which was due to the shrinkage of the socks and their full relaxation. The structural changes also influenced the higher elasticity of the tested specimens, which were uniformly stretched over a flattened rubber surface of Martindale specimen holders. This was supported by the fact that most of the pretreated sock samples in group B made from a higher percentage of elastic PA yarn (Table 3) showed a significant increase in the measured endpoints (compared to socks in group A).

According to the EN ISO 12945-4:2020, a pill is defined as the entangling of fibers into balls that protrude from the fabric and are of such a density that light cannot penetrate it and it casts a shadow. This change may occur during washing and/or wearing. In the pretreated sock samples, no pilling was observed on the surface after the five repeated washing and drying cycles. However, during the wear simulation, the propensity to surface pilling increased with the increasing number of abrasion cycles for all the untreated and pretreated sock plain knits, as shown in Table 6.

The best rated untreated plain knits were in sock group A, especially those made from air-jet spun modal and micro modal yarns, as they were less hairy, specifically regular and densely structured when compared to ring and rotor spun yarns (Figure 2). These sock plain knits showed only partially formed pills on their surface after 7000 pilling rubs (grade 4 and 4/5). Within sock group A, the pilling tendency of the conventional cotton socks was higher than that of the other tested socks. The results presented in Table 6 show that the coarser polyamide plating yarn PA 6.6 (2) used in the socks in groups B and C, as well as the cotton spun yarn CO -Ri (2) used in the socks in group C, had a negative effect on the tendency of the sock plain knits for surface pilling, resulting in a reduction in the pilling grades.

After 7000 pilling rubs, lower final grades were found for all the tested pretreated sock knit samples, as shown numerically in Table 6 and visually in Table 7 for the MMD-Ai sock samples. The best grades (2, 2/3 and 3), and thus, a lower propensity to surface piling after the five consecutive domestic washing and drying cycles, were exhibited by the sock knits made from the highest percentage of rotor spun modal and micro modal yarns. These pretreated sock knits exhibited moderate and distinct pilling on their surfaces and, in general, the propensity to surface pilling was less than that of conventional cotton socks. These results of the wear resistance obtained by both tests confirm the justification of the proposed evaluation methodology and the tests performed on the sock samples after simulating domestic care.

**Figure 13:**
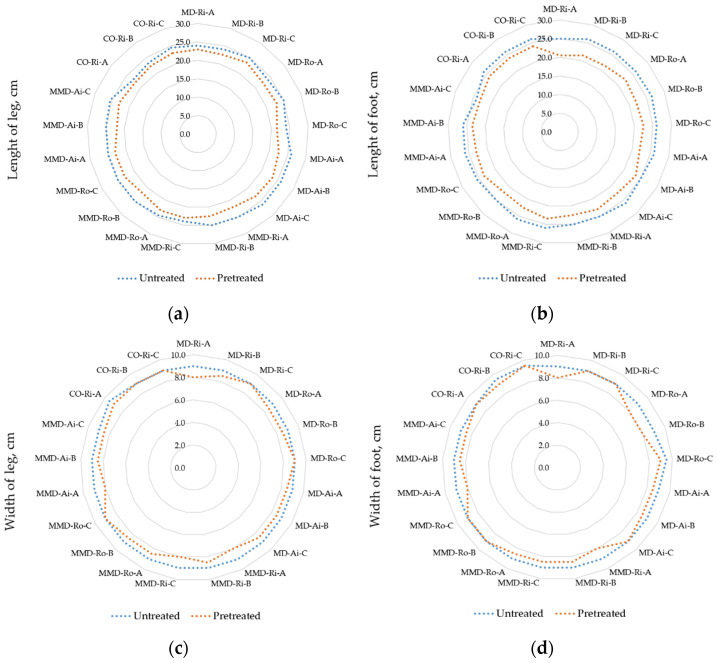
Measured sock dimensions: (**a**) length of the leg part; (**b**) length of the foot part; (**c**) width of the leg part; (**d**) width of the foot part of the untreated and pretreated socks determined before and after five repeated washing and drying cycles (where MD—modal fibers, MMD—micro modal fibers, CO—cotton fibers; Ri—ring spun yarn, Ro—rotor spun yarn, Ai—air-jet spun yarn; A, B, C—sock group).

Socks tend to change their dimensions greatly with repeated washing and drying. In this study, the dimensional changes were observed in the leg and foot length and the leg and foot width of the socks. Major changes were observed after the first pretreatment cycle and remained almost unchanged after five cycles. Therefore, the dimensions measured before and after the five repeated washing and drying cycles in the length and width directions are shown in Figure 13, and the calculated percentage change is shown in Table 8.

From the results shown in Figure 13, it can be seen that the measured dimensions decreased after the five pretreatment cycles for all the sock samples tested, resulting in the shrinkage in both the length and width. The difference in the dimensions between the untreated and pretreated socks, was higher in the lengthwise direction of the socks, as shown in Table 8. The results obtained could be related to the increase in the density values of the pretreated sock plain knits discussed in Section 3.1. The calculated shrinkage of the sock leg length ranged from 4.17% to 13.46%, with the lowest values obtained for the sock samples MD-Ri-A and MMD-Ri-C and the highest for the sock sample MMD-Ai-A. In general, the determined shrinkage was lower and comparable for the leg length of the socks made from the highest percentage of modal and cotton ring yarns (MD-Ri and Co-Ri). The shrinkage of the sock foot length ranged from 4.17% to 18.00% for the MMD-Ai-C and MD-Ri-A sock samples, respectively.

The leg and foot width of the socks measured in several samples remained unchanged after the pretreatment. In most cases, these were the heaviest and thickest socks in group C, as shown in Table 5. Since the elasticity of the sock plain knits depends primarily on the elasticity of the yarn used for the plating, it was found that increasing the percentage of polyamide (in sock groups B and C) mainly reduces the shrinkage of the socks in the width direction. Since the higher values of the transverse elasticity and stable width of the socks provide easier wearing, the present test results point in favor of this.

As the sock construction allows for shrinkage of up to 40% [5], it could be concluded that the dimensional stability of the socks is satisfactory and is strongly influenced by their physical properties and the fiber and yarn blend used for their production.

### 3.3. Socks Comfort Properties

**Figure 14:**
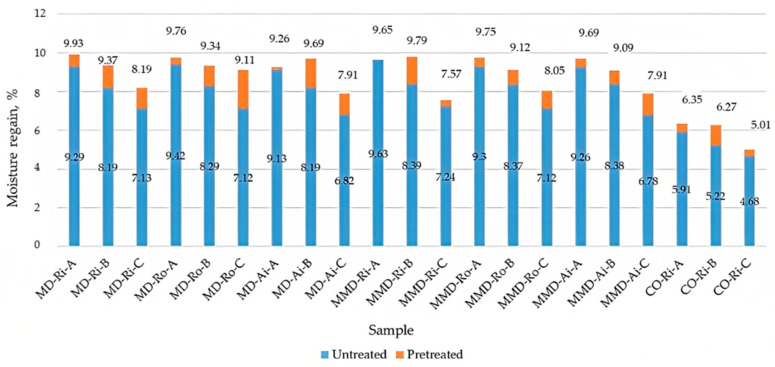
Moisture regain of the untreated and pretreated sock plain knit samples determined before and after five repeated washing and drying cycles of the socks (where MD—modal fibers, MMD—micro modal fibers, CO—cotton fibers; Ri—ring spun yarn, Ro—rotor spun yarn, Ai—air-jet spun yarn; A, B, C—sock group).

**Figure 15:**
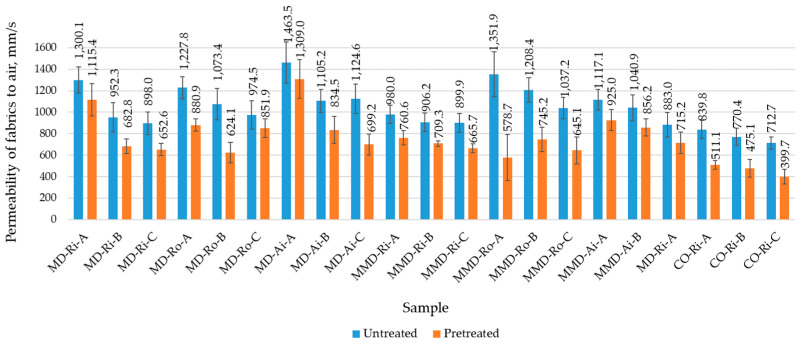
Air permeability of the untreated and pretreated sock plain knit samples determined before and after five repeated washing and drying cycles of the socks (where MD—modal fibers, MMD—micro modal fibers, CO—cotton fibers; Ri—ring spun yarn, Ro—rotor spun yarn, Ai—air-jet spun yarn; A, B, C—sock group).

**Figure 16:**
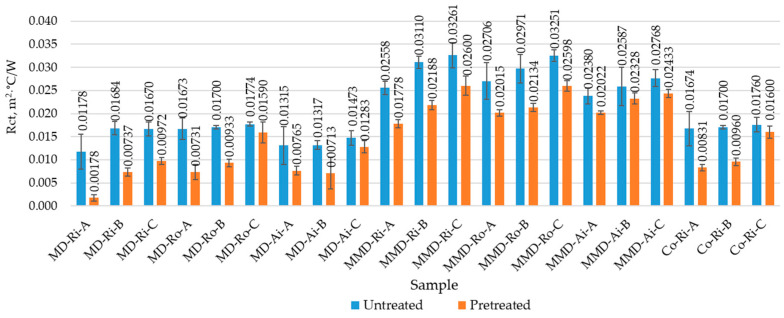
Thermal resistance (Rct) of the untreated and pretreated socks determined before and after five repeated washing and drying cycles (where MD—modal fibers, MMD—micro modal fibers, CO—cotton fibers; Ri—ring spun yarn, Ro—rotor spun yarn, Ai—air-jet spun yarn; A, B, C—sock group).

The results of the measured comfort-related properties of the untreated and pretreated sock samples are shown in Figure 14, Figure 15 and Figure 16. Table 9 contains a comparison of the mean values of the moisture regain, air permeability and thermal resistance of the untreated and pretreated socks (made from differently spun modal or micro modal yarn) calculated for groups A, B and C with the corresponding standard deviation.

From the results shown in Figure 14, it can be seen that the determined moisture regain decreased for the thicker and heavier sock plain knits. For the untreated sock samples, the highest values were found in sock group A and the lowest in sock group C. Within the same group of the untreated socks made from the highest percentage of differently spun modal yarns (Table 9), the moisture regain mean value for sock group A was 9.28%, for sock group B was 8.22% and for sock group C was 7.02%. For the socks made from differently spun micro modal yarns, the mean values of the moisture regain were 9.40%, 8.38% and 7.05% for groups A, B and C, respectively, with the lowest values found in the socks made from air-jet spun yarns. In the untreated conventional cotton socks made from ring spun yarns, the water vapor absorption was lower, ranging from 5.91% to 4.68% (Figure 14). Despite the minor variation within the sock groups, the slightly higher mean values for the moisture regain were calculated for the same group of socks (A, B and C) made from micro modal yarns (Table 9). This suggests that a more uniform structure of the micro modal yarns (as discussed in Section 3.1), which has a major influence on the slightly lower mean values of the determined sock knit thickness (Table 5), also influences the higher mean values of the moisture regain determined for the same group of socks (Table 9).

In support of this finding, it was found that, with the increase in the polyamide content in sock groups B and C and the cotton content in group C, the moisture regain values decreased. The differences found between the fiber types were related to the hygroscopicity of the fibers. Modal fibers provided better hydrophilicity than cotton, as shown in Table 1. When measuring the moisture absorption, the fiber type had the greatest influence, as the sock knits made from more hydrophobic polyamide fibers absorbed less moisture than the knits made from the two types of modal, which were hydrophilic. Any difference in the behavior of the moisture properties between the modal and micro modal fibers may be due to the morphology of the fibers and yarns since their affinity for water was the same.

The influence of the structure of the plain knits on the investigated properties was confirmed after the wet pretreatment of the socks, during which the water vapor absorption increased in all the tested sock samples (Figure 14). It was found that, in the pretreated socks, the moisture regain increased up to 27% for the socks made from modal fibers and up to 16.8% for the socks made from micro modal fibers and cotton. The highest increase was found in the sock plain knits in groups B and C (Table 9). This can also be explained by the full relaxation of the socks and the presence of the structural differences from which they provide a better ability for absorbing moisture (as explained in Section 3.1 and Section 3.2).

The higher determined values of the water vapor absorption certainly have an effect on the feeling of the higher wearing comfort due to sweating. However, when the fiber swells, the free space in the sock decreases and so does the transport of sweat from the inside to the outside. Therefore, the air permeability is an important comfort factor to transport moisture away from the skin and ensure the breathability of the socks. Since the air permeability also depends on the thickness and surface porosity of the sock knits [33], the results shown in Figure 15 indicate that all the untreated sock samples in group A had the highest values for the air permeability and, thus, the highest porosity and breathability. Increasing the thickness and mass of the sock knits, i.e., the tightness and compactness of their structure, prevented the passage of air and reduced the air permeability in sock groups B and C.

As can be seen in Figure 15 and Table 9, the air permeability results showed a higher variability within the measurements for the sock samples, especially within the same sock group made from differently spun modal and micro modal yarns. This was confirmed by the fact that, in the group of the untreated socks made from modal yarns, the values of the air permeability in sock group A ranged from 1227.8 mm/s to 1463.5 mm/s, in sock group B from 952.3 mm/s to 1208.4 mm/s and in sock group C from 898.0 mm/s to 1124.6 mm/s, with the highest values found for the socks made from air-jet spun yarns. For the untreated socks made from micro modal yarns, the air permeability values in sock group A ranged from 980.0 mm/s to 1351.9 mm/s, in sock group B from 906.2 mm/s to 1105.2 mm/s and in sock group C from 883.0 mm/s to 1037.2 mm/s, with the highest values found for the socks made from rotor spun yarns and the lowest for the socks made from ring spun yarns. In the untreated conventional cotton socks made from ring spun yarns, the permeability of air was lower and in the range of 712.7 mm/s to 839.8 mm/s (Figure 15).

It was found that the socks made from spun yarns with hairy surfaces, such as ring spun yarns, primarily have a lower air permeability than the socks made from rotor and especially air-jet yarns with more uniform surfaces, as shown in Figure 2 and discussed in Section 3.1. This is due to the fact that, as the hairiness of the yarn surface decreases, the protruding fibers become more firmly attached to the yarn and channels (open pores) open up between the neighboring yarn loops in the same area of the sock knit. The untreated socks made from modal yarns are mainly more breathable than the socks made from micro modal yarns, probably due to the different fineness of the fibers and their packing density in the yarns used for knitting. The untreated conventional cotton socks made from ring spun yarns are less breathable compared to MD and MMD socks. This can also be partially attributed to the convolutions in the cotton fibers and their surface properties, as well as the non-smooth cross-sections, as shown in Table 1, that impede the passage of air through the fabric.

After the five repeated washing and drying cycles, the air permeability of all the tested socks plain knits was significantly reduced due to the shrinkage and relaxation of the sock structure (Table 9). It was found that the air permeability of the pretreated socks decreased up to 72% for the socks made from modal yarns, up to 130% for the socks made from micro modal yarns and up to 78% for cotton socks. Reducing the air permeability can be an advantage in colder months. However, since all the socks were elastic due to the addition of plating a polyamide multifilament yarn in each row of knitting, they stretch at the foot of the wearer and the open porosity and breathability of the socks are increased, which can have a positive effect on wearing comfort.

The results of the thermal resistance of the untreated and pretreated socks, determined as the arithmetic mean of the three individual sock measurements with a corresponding standard deviation, are shown in Figure 16.

As presented in Table 5, the mean values of the thickness calculated in groups A, B and C of the untreated socks made from differently spun modal yarns (0.90 mm, 0.98 mm and 1.04 mm, respectively) were minimally higher than the same values of the sock groups made from differently spun micro modal yarns (0.89 mm, 0.96 mm and 1.00 mm).

From the results presented in Table 9, it is clear that the socks in group A had the lowest mean values of the thermal resistance. As the thickness and mass of the socks increased (Table 5), as well as the tightness of their structure, which prevents the passage of heat, the thermal resistance in sock groups B and C increased. It was confirmed that the volume of trapped air determines the ability of the socks to resist the heat flow through their knitted structure. Thinner knits trap less air and are, therefore, less insulating than thicker knits.

The effect of the fiber fineness on the thermal resistance was mostly limited to the packing density in the yarn structure, which contributed to the volume of trapped air. The literature [23] indicated that fabrics made from microfibers have lower thermal conductivity and, thus, better insulation properties. Since micro modal yarns used for the knitting of socks contain a higher number of fibers per unit length than modal yarns, their packing density and surface area are higher. This resulted in higher thermal resistance values for the sock groups with the highest percentage of micro modal yarns compared to those made from modal yarns (Table 9), although the mean thickness values were lower in the MMD sock groups (Table 5).

However, as Figure 16 shows, the thermal resistance varies even within the same sock group. The sock samples made from modal rotor spun yarns (in sock groups A, B and C) had higher thermal resistance values than the socks made from ring and air-jet spun yarns. The finding that the overall unevenness of rotor spun modal and micro modal yarns was higher compared to ring and air-jet spun yarns (as discussed in Section 3.1 and [27]) led to the conclusion that the uneven structure of rotor spun yarns, especially in the outer layer (Figure 2), and the kitted structure produced from them contains a larger volume of air and, therefore, has a major influence on the obtained results. It should also be noted that, in the groups of the untreated socks made from modal and micro modal yarns, the lowest values of thermal resistance were found primarily in the socks made from air-jet spun yarns with the lowest hairiness. This can be related to the previously discussed results of the determined air permeability, as shown in Figure 15. It follows that the sock samples with a higher air permeability and, thus, a higher porosity offer less resistance to the heat flow.

The thermal resistance of conventional cotton socks made from ring spun yarns was higher than that of the socks made from modal ring spun yarns. This could be due to the fact that cotton socks have a slightly thicker structure and, thus, a lower water vapor absorption and air permeability, as shown in Figure 10, Figure 14 and Figure 15.

The foot length of the untreated socks was approx. 20% shorter than the length of the wearer’s foot and, after the pretreatment of the socks, it was additionally shortened up to 18%. Due to the observed shrinkage of the pretreated socks, as shown in Table 8, the socks were additionally stretched during the test on the thermal foot (as well as on the wearer’s foot), which led to an increase in the porosity of their structure and, thus, to a higher breathability of the socks. This can have a positive effect on the wearing comfort in warmer weather and is reflected in the significantly lower resistance to the passage of heat in all the tested sock samples after the five repeated pretreatment cycles (Figure 16, Table 9).

## 4. Conclusions

The study described in this paper aimed to determine the applicability of modal and micro modal yarns for the manufacture of socks, to investigate the influence of the differently spun main yarn types and the polyamide plating yarns used on the physical usage and comfort properties of socks and to confirm the proposed methodology for sock evaluation. Three groups of socks were designed—groups A and B with three single spun modal or micro modal yarns of the same type (ring, rotor or air-jet) and linear density, and group C where one of these yarns was replaced by a coarser cotton ring spun yarn. The socks were knitted in a single jersey pattern in full plating with different polyamide 6.6 yarns and compared to conventional cotton socks. From the results presented, the following can be concluded.

The socks samples in group A made from MD and MMD yarns had a lower weight than the socks in groups B and C, with almost no variation within the same group and in comparison with cotton socks. The coarser polyamide plating yarn used in sock groups B and C and the cotton yarn in group C changed their structure, and the socks became thicker and heavier. The more uniform structure of the MMD yarns had a major impact on the slightly lower thickness values obtained in the MMD sock groups. The mass per unit area showed a greater variability within and between the same sock groups due to the differences in the yarn and sock knit structure. After the five repeated washing and drying cycles, the sock weight remained almost the same for cotton socks, while a minimal increase of up to 0.5% was observed for the socks made from MD and MMD yarns. Due to the shrinkage potential, the socks became more voluminous and had higher density, resulting in an increase in the mass per unit area and thickness of up to 16%.

The untreated sock knits made from modal ring spun yarns had a better abrasion resistance than those made from rotor and air-jet modal spun yarns. The untreated MD group A sock samples exhibited the highest abrasion resistance. Although the polyamide content was increased in sock groups B and C, no improvement in the abrasion resistance was observed. The abrasion resistance was lower in the untreated MMD socks, with sock group B (made from ring and rotor spun yarns) achieving better results. The abrasion resistance increased greatly after the five repeated pretreatment cycles for all the sock samples tested—up to 77% for MMD socks, up to 80% for MD socks and up to 56% for cotton socks. Overall, the abrasion resistance of the pretreated MD socks and, in most cases, the MMD socks was comparable to that of conventional cotton socks, with significant thinning occurring in the interval from 30,000 to 45,000 abrasion rubs.

The group A untreated socks exhibited the best pilling resistance, especially those made from air-spun MD and MMD yarns. These untreated socks showed only partially formed pills on the surface after 7000 pilling rubs. Within sock group A, the pilling tendency was higher in the conventional cotton socks. The polyamide plating yarn used in sock groups B and C and the cotton yarn used in the group C socks had a negative influence on the obtained results. Lower final grades were found in all the tested pretreated socks, with the best grades obtained by the MD and MMD socks made from rotor spun yarns.

After the five pretreatment cycles, the shrinkage in the lengthwise direction of the socks was higher in all the tested socks. The determined shrinkage in the leg and foot length was maximum 13.5% and 18.0%, respectively. The determined shrinkage was lower in leg length for the socks made from MD and CO ring yarns. Increasing the polyamide content (in sock groups B and C) primarily reduced the shrinkage of the socks in the width direction.

The moisture regain decreased for the thicker and heavier socks in groups B and C, with the lowest values found for the MD and MMD socks made from air-jet spun yarns. Slightly higher mean values for the moisture regain were calculated for all the MMD sock groups. With the increase in the polyamide content in sock groups B and C and the cotton content in group C, the values for the moisture regain values decreased, since modal fibers have a better hydrophilicity than cotton and PA. After the wet pretreatment, the water vapor absorption increased in all the tested socks—up to 27% in MD socks and up to 16.8% in MMD and cotton socks, which can also be explained by the full relaxation of the socks and the presence of the structural differences from which they provided a better ability for absorbing moisture.

Increasing the thickness and mass of the sock knits, i.e., the tightness and compactness of their structure, decreased the air permeability in sock groups B and C compared to group A. The results showed a greater variability within the same sock groups, with the highest values found in the untreated MD socks for the socks made from more uniform air-jet spun yarns. The untreated MMD socks had a lower air permeability, with the highest values found for the socks made from rotor yarn and the lowest for the socks made from ring spun yarn with a hairy surface. The untreated MMD socks were more breathable, primarily due to the different fineness of the fibers and their packing density in the yarns used for knitting. The untreated cotton socks were less breathable compared to the MD and MMD socks. The pretreated socks had a significantly lower air permeability—up to 72% for MD socks, up to 130% for MMD socks and up to 78% for cotton socks.

The socks in group A had the lowest mean thermal resistance values. As the thickness and mass of the sock knits increased, the thermal resistance in sock groups B and C increased. The packing density and surface area of MMD yarns are higher, resulting in a higher thermal resistance in the MMD socks, although their average thickness was lower compared to the MD socks. The MD sock samples from rotor spun yarns with a more uneven structure had higher thermal resistance values. The lowest thermal resistance values were found primarily in the socks made from air-jet spun yarns with the lowest hairiness. Due to the observed shrinkage, the pretreated socks were additionally stretched during the test, which was reflected in the significantly lower thermal resistance.

The results obtained in all the tests confirmed the justification of the proposed evaluation methodology and the tests performed on the sock samples after the simulated domestic care.

The applicability of unconventionally spun modal and micro modal yarns in knitting socks was confirmed through the comparison with conventional cotton socks. It is expected that the outcome of this study will be used in the selection of fibers and yarns for the production of socks with specific properties. Future studies will investigate the applicability of the various innovative blends of viscose and lyocell fibers and the yarns made from them using the unconventional spinning processes for sock knitting.

## Figures and Tables

**Table 1:** Properties comparison of cotton and modal staple fibers [6,7,8].

Fiber Type	Cotton	Modal
Elongation at break (%) - conditioned - wet		
	8–10	10–15
	12–14	11–16
Tenacity (cN/tex) - conditioned - wet		
	25–30	36–42
	26–32	27–30
Moisture regain (%)	7.0–9.5	11.5–12.5
Water retention value (%)	42–53	55–70
Micrographs of the fiber cross and longitudinal view (scanning electron microscopy) *	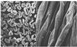	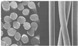

* For cotton and modal fibers images [9].

**Table 2:** Properties of the yarns used in the knitting of socks.

Yarn Type	Linear Density, Tex	Breaking Force, cN	Breaking Elongation, %	Tenacity, cN/Tex	Work of Rupture, cNcm
Main yarn *					
MD-Ri	20.0	487 ± 10	10.2 ± 0.2	24.3 ± 0.5	1436 ± 47
MD-Ro	20.0	325 ± 9	7.2 ± 0.2	16.3 ± 0.5	738 ± 32
MD-Ai	20.0	406 ± 10	9.0 ± 0.2	20.3 ± 0.5	1067 ± 42
MMD-Ri	20.0	506 ± 11	9.5 ± 0.2	25.3 ± 0.6	1421 ± 50
MMD-Ro	20.0	344 ± 10	7.3 ± 0.2	17.2 ± 0.5	777 ± 42
MMD-Ai	20.0	365 ± 11	8.2 ± 0.2	18.2 ± 0.5	886 ± 46
CO-Ri (1)	20.0	302 ± 5	3.7 ± 0.1	15.1 ± 0.3	301 ± 10
CO-Ri (2)	25.0	326 ± 8	3.8 ± 0.1	13.3 ± 0.3	333 ± 15
Plating yarn *					
PA 6.6 (1)	15.6	652 ± 8	26.07 ± 0.6	41.8 ± 0.5	4775 ± 180
PA 6.6 (2)	22.0	991 ± 4	28.5 ± 0.2	45.0 ± 0.2	7846 ± 81
Lycra	54.0	551 ± 14	321.0 ± 18	10.2 ± 0.4	2467 ± 324

* MD—modal fibers, MMD—micro modal fibers, CO—cotton fibers, PA 6.6—polyamide fibers; Ri—ring spun yarn, Ro—rotor spun yarn, Ai—air-jet spun yarn.

**Table 3:** Specification and structure characteristics of the socks produced.

Sock Group	Yarn Type		Fiber Content, %	
			Leg and Foot/ Plain Pattern	Cuff/ Rib Pattern
A	Main yarn *	Modal: Ri, Ro or Ai × 3 Micro modal: Ri, Ro or Ai × 3 Cotton: Ri (1) × 3	79 ± 1	55 ± 1
	Plating yarn	Polyamide 6.6 (1) × 1 Lycra × 1	21 ± 1 -	14 ± 1 31 ± 1
B	Main yarn *	Modal: Ri, Ro or Ai × 3 Micro modal: Ri, Ro or Ai × 3 Cotton: Ri (1) × 3	71 ± 1	52 ± 1
	Plating yarn	Polyamide 6.6 (2) × 1 Lycra × 1	29 ± 1 -	19 ± 1 29 ± 1
C	Main yarn *	Modal: Ri, Ro or Ai × 2 Micro modal: Ri, Ro or Ai × 2 Cotton: Ri (1) × 2	44 ± 1	42 ± 1
		+ Cotton: Ri (2) × 1	28 ± 1	18 ± 1
	Plating yarn	Polyamide 6.6 (2) × 1 Lycra × 1	28 ± 1 -	16 ± 1 24 ± 1

* Ri—ring spun yarn, Ro—rotor spun yarn, Ai—air-jet spun yarn.

**Table 4:** The untreated and pretreated sock plain knit samples’ density parameters (number of wales per cm, courses per cm and stitches per cm^2^) determined before and after five repeated washing and drying cycles of the socks.

Sock Sample *	Wales, cm^−1^		Courses, cm^−1^		Stitches, cm^−2^	
	Untreated	Pretreated	Untreated	Pretreated	Untreated	Pretreated
MD-Ri-A	6	7	7	8	42	56
MD-Ri-B	6	7	7	9	42	63
MD-Ri-C	6	6	7	8	42	48
MD-Ro-A	6	6	8	9	48	54
MD-Ro-B	6	6	8	9	48	54
MD-Ro-C	6	6	7	9	42	54
MD-Ai-A	6	6	7	9	42	54
MD-Ai-B	6	6	7	8	42	48
MD-Ai-C	6	6	7	8	42	48
MMD-Ri-A	6	7	8	8	48	56
MMD-Ri-B	6	7	8	8	48	56
MMD-Ri-C	6	6	8	8	48	48
MMD-Ro-A	6	6	8	9	48	54
MMD-Ro-B	6	7	7	8	42	56
MMD-Ro-C	6	6	7	8	42	48
MMD-Ai-A	6	6	8	9	48	54
MMD-Ai-B	6	6	7	9	42	54
MMD-Ai-C	6	6	7	8	42	48
CO-Ri-A	6	6	8	9	48	54
CO-Ri-B	6	6	8	9	48	54
CO-Ri-C	6	6	8	8	48	48

* MD—modal fibers, MMD—micro modal fibers, CO—cotton fibers; Ri—ring spun yarn, Ro—rotor spun yarn, Ai—air-jet spun yarn; A, B, C—sock group.

**Table 5:** Mean values of the physical properties calculated for groups A, B and C of the untreated and pretreated socks made from differently spun modal and micro modal yarns.

Sock Group *	Weight of Sock, g		Mass per Unit Area, g/m^2^		Thickness, mm	
	Untreated	Pretreated	Untreated	Pretreated	Untreated	Pretreated
MD-A	20.1 ± 0.14	20.1 ± 0.16	264.0 ± 2.86	300.6 ± 11.43	0.90 ± 0.021	1.03 ± 0.034
MD-B	22.4 ± 0.16	22.5 ± 0.17	284.6 ± 3.86	311.4 ± 12.45	0.98 ± 0.022	1.09 ± 0.012
MD-C	23.8 ± 0.04	23.8 ± 0.05	302.7 ± 7.41	321.6 ± 9.37	1.04 ± 0.021	1.13 ± 0.012
MMD-A	20.0 ± 0.22	20.1 ± 0.25	271.9 ± 3.37	298.2 ± 2.16	0.89 ± 0.025	0.98 ± 0.028
MMD-B	22.4 ± 0.26	22.5 ± 0.25	283.6 ± 5.63	305.8 ± 9.21	0.96 ± 0.005	1.02 ± 0.014
MMD-C	23.8 ± 0.17	23.8 ± 0.17	306.8 ± 5.70	336.8 ± 13.33	1.00 ± 0.017	1.11 ± 0.009

* MD—modal fibers, MMD—micro modal fibers, A, B, C—sock group.

**Table 6:** Visually assessed propensity to surface pilling in the untreated and pretreated sock plain knits using grades of pilling.

Sock Sample *	Untreated						Pretreated					
	Number of Pilling Rubs											
	125	500	1000	2000	5000	7000	125	500	1000	2000	5000	7000
MD-Ri-A	4/5	4/5	4/5	4	4	3/4	4/5	4	3	2/3	2	2
MD-Ri-B	4	4	3/4	3/4	3	2/3	4/5	4/5	4	3/4	3	1
MD-Ri-C	3/4	3	2/3	2	1	1	4/5	4	3	2/3	2	1
MD-Ro-A	4/5	4/5	4/5	4	4	3	4/5	4/5	4	3/4	3	2/3
MD-Ro-B	4/5	4	4	3/4	2/3	2/3	4/5	4/5	4	4	3/4	3
MD-Ro-C	4	3/4	3	2/3	2	1	4/5	4/5	4	3/4	3/4	3
MD-Ai-A	5	5	5	5	4/5	4/5	4/5	3/4	3	2/3	2	1
MD-Ai-B	4/5	4/5	4/5	3/4	3	2/3	4/5	3	2/3	2	1	1
MD-Ai-C	4	3/4	3	3	1/2	1	4/5	4/5	3/4	3	2/3	2
MMD-Ri-A	5	5	5	5	4/5	4	4	3	2	2	1/2	1
MMD-Ri-B	5	4/5	4/5	4/5	4	2/3	4/5	3	2/3	2	1/2	1
MMD-Ri-C	3/4	3	2/3	2/3	2	1	4/5	4	3	2/3	2	1
MMD-Ro-A	5	4/5	4/5	4/5	4	3	4/5	4	3/4	3	2/3	2
MMD-Ro-B	5	4/5	4/5	4/5	4	3	4/5	4	3/4	3	2/3	2
MMD-Ro-C	3	2/3	2	1/2	1	1	4/5	4	3	2/3	2	1
MMD-Ai-A	5	5	5	5	4/5	4	4/5	3/4	3	2/3	1/2	1
MMD-Ai-B	5	4/5	4/5	4/5	4	3/4	4/5	3	2/3	2	1	1
MMD-Ai-C	5	4/5	4	3/4	3	2/3	4/5	4	3/4	3	1	1
CO-Ri-A	4/5	4	3/4	3	3	2/3	5	4	3	2/3	1/2	1
CO-Ri-B	4/5	4	4	3/4	3	2/3	4/5	4	3	2/3	2	1/2
CO-Ri-C	4/5	4	3/4	3	3	2	4/5	4	3	3	2	1

* MD—modal fibers, MMD—micro modal fibers, CO—cotton fibers; Ri—ring spun yarn, Ro—rotor spun yarn, Ai—air-jet spun yarn; A, B, C—sock group.

**Table 7:** Surface appearance of the untreated and pretreated sock plain knits made from micro modal air-jet spun yarns (MMD-Ai) at the end of the pilling test.

Sock Sample	MMD-Ai-A	MMD-Ai-B	MMD-Ai-C
Untreated *	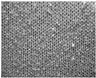	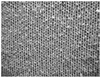	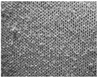
No. Rubs/Pilling Grade	7000/4	7000/3–4	7000/2–3
Pretreated *	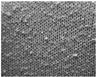	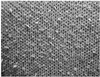	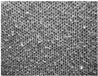
No. Rubs/Pilling Grade	7000/1	5000/1	5000/1

* Optical microscopy images taken with a magnification of 10×.

**Table 8:** Dimensional stability of the socks after five pretreatment cycles.

Sock Sample *	Changes in Dimension, %			
	Leg Length	Foot Length	Leg Width	Foot Width
MD-Ri-A	−4.17	−18.00	−11.112	−11.11
MD-Ri-B	−6.25	−17.31	−5.56	0.00
MD-Ri-C	−6.00	−17.31	0.00	0.00
MD-Ro-A	−6.25	−13.46	−5.56	−11.11
MD-Ro-B	−8.00	−16.98	−5.56	−11.11
MD-Ro-C	−10.41	−13.46	0.00	−5.26
MD-Ai-A	−13.46	−15.38	−5.56	−5.56
MD-Ai-B	−9.61	−6.00	−5.56	−5.56
MD-Ai-C	−11.53	−13.46	−5.56	0.00
MMD-Ri-A	−12.00	−8.00	−11.11	−11.11
MMD-Ri-B	−10.00	−10.00	−5.56	−5.56
MMD-Ri-C	−4.17	−9.61	−11.11	−5.56
MMD-Ro-A	−6.12	−13.46	−5.56	−5.56
MMD-Ro-B	−12.00	−12.00	−5.56	0.00
MMD-Ro-C	−8.00	−7.84	0.00	0.00
MMD-Ai-A	−8.00	−11.53	−11.11	−11.11
MMD-Ai-B	−12.00	−9.61	−5.56	5.56
MMD-Ai-C	−9.80	−4.17	−5.56	−5.56
CO-Ri-A	−4.35	−7.69	−5.56	0.00
CO-Ri-B	−4.25	−7.69	0.00	−5.26
CO-Ri-C	−6.12	−7.69	0.00	0.00

* MD—modal fibers, MMD—micro modal fibers, CO—cotton fibers; Ri—ring spun yarn, Ro—rotor spun yarn, Ai—air-jet spun yarn; A, B, C—sock group.

**Table 9:** Mean values of the comfort properties calculated for groups A, B and C of the untreated and pretreated socks made from differently spun modal and micro modal yarns.

Sock Group *	Moisture Regain, %		Air Permeability, mm/s		Thermal Resistance, m^2^∙C/W	
	Untreated	Pretreated	Untreated	Pretreated	Untreated	Pretreated
MD-A	9.28 ± 0.119	9.65 ± 0.284	1330.47 ± 98.59	1101.77 ± 175.04	0.01389 ± 0.00209	0.00558 ± 0.00269
MD-B	8.22 ± 0.047	9.47 ± 0.158	1043.63 ± 65.87	713.80 ± 88.65	0.01567 ± 0.00177	0.00794 ± 0.00099
MD-C	7.02 ± 0.144	8.40 ± 0.513	999.03 ± 94.12	734.57 ± 85.12	0.01639 ± 0.00125	0.01282 ± 0.00252
MMD-A	9.40 ± 0.166	9.70 ± 0.041	1149.67 ± 153.56	754.77 ± 141.44	0.02548 ± 0.00133	0.01938 ± 0.00113
MMD-B	8.38 ± 0.008	9.33 ± 0.323	1051.83 ± 123.61	770.23 ± 62.53	0.02889 ± 0.00221	0.02217 ± 0.00082
MMD-C	7.05 ± 0.195	7.84 ± 0.202	940.03 ± 69.05	675.33 ± 29.42	0.03093 ± 0.00230	0.02544 ± 0.00078

* MD—modal fibers, MMD—micro modal fibers, A, B, C—sock group.

## Data Availability

Data available in a publicly accessible repository.
